# Açai Berry Attenuates Cyclophosphamide-Induced Damage in Genitourinary Axis-Modulating Nrf-2/HO-1 Pathways

**DOI:** 10.3390/antiox11122355

**Published:** 2022-11-28

**Authors:** Rosalba Siracusa, Ramona D’Amico, Roberta Fusco, Daniela Impellizzeri, Alessio Filippo Peritore, Enrico Gugliandolo, Rosalia Crupi, Livia Interdonato, Marika Cordaro, Salvatore Cuzzocrea, Rosanna Di Paola

**Affiliations:** 1 Department of Chemical, Biological, Pharmaceutical and Environmental Sciences, University of Messina, Viale Ferdinando Stagno D’Alcontres 31, 98166 Messina, Italy; 2 Department of Veterinary Sciences, University of Messina, 98168 Messina, Italy; 3 Department of Biomedical Dental and Morphological and Functional Imaging, University of Messina, Via Consolare Valeria, 98125 Messina, Italy; 4 Department of Pharmacological and Physiological Science, Saint Louis University School of Medicine, Saint Louis, MO 63104, USA

**Keywords:** Nrf2, oxidative stress, inflammation, cyclophosphamide, bladder, testes

## Abstract

Cyclophosphamide (CYP) is used to treat different malignancies and autoimmune disorders in men. This chemotherapy frequently reduces tumors, which is beneficial, but also causes infertility because of severe oxidative stress, inflammation, and apoptosis in the bladder and testes brought on by its metabolite, acrolein. The goal of this study was to assess the efficacy of a novel food, açai berry, in preventing CYP-induced damage in the bladder and testes. Methods: CYP was administered intraperitoneally once during the experiment at a dose of 200 mg/kg body weight diluted in 10 mL/kg b.w. of water. Açai berry was administered orally at a dose of 500 mg/kg. Results: The administration of açai berry was able to reduce inflammation, oxidative stress, lipid peroxidation, apoptosis, and histological changes in the bladder and testes after CYP injection. Conclusions: Our findings show for the first time that açai berry modulates physiological antioxidant defenses to protect the bladder and testes against CYP-induced changes.

## 1. Introduction

One of the most serious illnesses in the world is cancer, and researchers have tried to find strategies to stop it or enhance patients’ quality of life. A healthy lifestyle, which requires an adequate diet, is thought to be able to prevent more than two-thirds of human cancers [1]. Cyclophosphamide (CYP; N,N-bis(2-choloroethyl)tetrahydro-2H-1,3,2-oxazaphosphorin-2-amine-2-oxide) is an alkylating agent with excellent cytotoxic effects that is frequently used as an anticancer or immunosuppressive treatment [2,3,4]. Specifically, it is used as chemotherapy for the treatment of lymphoma, multiple myeloma, leukemia, prostate and breast cancer, neuroblastoma, and sarcoma [5,6].

Although it has a wide range of clinical applications, CYP has several side effects, including anorexia, vomiting, hair loss, and bladder hemorrhage. The most severe adverse effects can include a higher chance of developing cancer, miscarriage, allergic reactions, and pulmonary fibrosis [3,4,7]. Male and female infertility as well as premature menopause have both been linked to CYP, with the risk increasing with cumulative medication dosage and patient age. This type of infertility is typically transient, but it can also be permanent [5,6]. CYP is metabolized by cytochrome P450 into two unstable intermediates, 4-hydroxycyclophosphamide and aldophosphamide, and then into two stable toxic intermediates, phosphoramide mustard and acrolein [8,9]. Phosphoramide mustard prevents cell division by forming cross-linkages both between and within DNA strands at the guanine N-7 position. This is irreversible and leads to cell death [10].

Conversely, acrolein, a reactive aldehyde, possesses the ability to generate toxic reactive oxygen species (ROS) and subsequently affect surrounding tissue [11,12]. Multiple effects of ROS, including the inhibition of several enzymes, DNA and membrane damage, and lipid peroxidation, contribute to infertility [12,13]. Oxidative stress (OS) plays a major role in the pathogenesis of idiopathic male infertility [14]. In fact, elevated testicular oxidative stress is a major contributor to increased germ cell apoptosis and eventual hypo-spermatogenesis, making it a major factor in the etiology of male infertility. It can cause changes in the patterns of testicular microvascular blood flow and endocrine signaling [15]. Recent investigations have demonstrated that OS affects the gene expression of somatic cells and gametes as well as epigenetic markers. Epigenetic processes that have been demonstrated to influence gene expression include chromatin remodeling, histone changes, noncoding RNA production, and DNA methylation [14,16,17,18]. 

Superoxide dismutase (SOD), glutathione (GSH), catalase (CAT), heme oxygenase-1 (HO-1), and NAD(P)H dehydrogenase (quinone) 1 (NQO1), primary enzymes that the body uses to fight OS, are activated by nuclear transcription factor-erythroid 2 related factor (Nrf2) [19,20]. When the body is functioning normally, Nrf2 is found in the cytoplasm linked to its antagonist, Kelch-like ECH-associating protein 1 (Keap1). The antioxidant response element (ARE), a regulatory enhancer area found in gene promoters, is where Nrf2 sequentially attaches after being released from the Keap1–Nrf2 complex in response to ROS. As a result of this interaction, numerous genes for detoxifying and antioxidant enzymes are induced, protecting cells from oxidative stress and a variety of toxins [21,22]. Extensive lipid peroxidation and protein oxidation result from ROS production that is greater than the antioxidant response system’s ability to scavenge it, which leads to improper cellular function [23]. For instance, it has been proposed that Nrf2 expression can be lowered when spermatozoa are exposed to high amounts of ROS and is necessary for normal spermatogenesis and sperm-specific capabilities, including motility [24,25,26,27].

The scientific community has been increasingly supporting the idea that food can serve as medicine and that diets high in plant foods and low in processed foods can prevent or reduce the severity of many diseases [19,28,29,30,31,32,33,34,35,36,37]. It is imperative to carry out research to identify natural substances that can potentially protect against CYP-induced oxidative stress while also lowering chemotherapy-related toxicity.

Açai berry has recently piqued the interest of scientists. This berry offers several healthy nutritional benefits and may have potential medical applications. The fruit of the açai palm, *Euterpe oleracea*, which grows only in the Amazon, has a tart flavor. It is said to be a high-energy fruit and has been used by Amazonian Indians for millennia as a food source and a natural treatment for a number of ailments [38,39,40,41,42,43,44,45,46]. 

Because the pulp of açai fruit contains a significant amount of bioactive nutrients and phytochemicals, it has been the subject of much research. In addition to a variety of physiologically active phytochemicals, the composition of açai berry pulp also has high concentrations of mono- and polyunsaturated fatty acids, which are uncommon in most fruits and berries. Açai pulp contains phytochemicals such proanthocyanidins, anthocyanins, and other flavonoids. Additionally, phytochemical tests showed that açai fruit contains considerable amounts of anthocyanins, such as cyanidin, delphinidin, malvidin, pelargonidin, and peonidin, as well as other polyphenolics, such as luteolin, quercetin, dihydrokaempferol, and chrysoerial. Five forms of carotenoids—carotene, lycopene, astaxanthin, lutein, and zeaxanthin—are found in the pulp of açai fruit [47,48,49,50,51,52,53].

The bioactive ingredients of açai berry extract have a wide range of pharmacological advantages, including anti-inflammatory and anti-anxiety activity, by modifying oxidative stress, inflammation, autophagy, and Nrf2 expression in the hippocampus and frontal cortex [54,55,56,57,58,59,60,61,62]. To confirm the protective, anti-inflammatory, and antioxidant effects, more evidence is required. For this reason, we looked at the possible health benefits of açai supplementation and the molecular mechanism by which it functions using a well-known experimental model of CYP-induced toxicity in the genitourinary axis.

## 2. Materials and Methods

### 2.1. Animals 

For this experiment, 8-week-old, 18 to 24 g male CD1 mice were purchased from Envigo (Milan, Italy), put in a controlled environment, and given free access to water and normal rodent food. They were kept in a 12:12 h light–dark cycle at 21.1 °C and 50.5% humidity in cages with five mice each. The University of Messina Review Board for animal care approved the study.

### 2.2. Experimental Design and Groups

We employed a validated mouse model, using a single intraperitoneal (i.p.) injection of CYP (200 mg/kg b.w) in distilled water (10 mL/kg b.w) to induce cystitis and testicular damage [19,36,63,64] (See Supplementary Material Figure S1 for a graphic illustration of the experimental design). 

After CYP injection, animals were randomly split into three groups:

(1) Sham: animals were administered injections of saline and treated orally with açai berry dissolved in saline;
(2) CYP: animals were administered CYP injections as described above and treated by oral gavage with saline;
(3) CYP+Açai Berry: animals were administered CYP injections as described above and treated with açai berry dissolved in saline (500 mg/kg) by oral gavage 1 h after injection and for the following 5 days.

At the end of the experiment, animals were anesthetized with ketamine (2.6 mg/kg) and xylazine (0.16 mg/kg) and subsequently beheaded. The bladder, testes, blood, sperm, and serum were collected. The dose of açai berry was calculated based on our previously published works [65,66,67].

### 2.3. Evaluation of Sperm

To obtain the sperm, the entire mouse epididymis was minced in a sperm-washing medium and incubated for 30 min at room temperature. The sperm parameters were evaluated as previously described in our other work [63,68]. To count the sperm, we used a Neubauer hemocytometer with 20 μL of sperm suspension. One drop of sperm suspension was placed on a slide to measure the percentage of sperm motility, which was then assessed under a light microscope at a magnification of 10× in three fields for each sample, and the proportion of sperm with normal and abnormal motility in each field was recorded. One drop of sperm suspension and 10 mL of eosin were mixed together to assess the percentage of sperm morphology. A drop of 12 μL of the prepared sample was smeared onto a glass slide after 1 min of incubation. Sperm morphology was evaluated after drying. The prepared slides were examined for abnormal sperm head and tail shapes, and mean values were taken. After 20 μL of sperm suspension was mixed with 20 μL of 1% eosin-Y, stained and unstained cells were counted using a Neubauer hemocytometer and an inverted microscope at a magnification of 40×. At least 3 measurements were taken on each sample. The sperm characteristics were determined according to the guidelines of the World Health Organization (WHO) in the WHO Laboratory Manual for the Examination and Processing of Human Semen, 2010. These are considered valid and are also used for evaluating animal sperm.

### 2.4. Western Blot Analysis of Cytosolic and Nuclear Extracts

Cytosolic and nuclear extracts were prepared from the bladder and testes as previously described [69,70]. The following primary antibodies were used: anti-NRF-2 (1:500, Santa Cruz Biotechnology, Heidelberg, Germany, #sc-365949), anti-caspase 3 (1:500, Santa Cruz Biotechnology, Heidelberg, Germany, #sc-7272), anti-heme oxygenase 1 (HO-1; 1:500, Santa Cruz Biotechnology, Heidelberg, Germany, #sc-136960), anti-Bax (1:500, Santa Cruz Biotechnology, #sc7480), and anti-Bcl-2 (1:500, Santa Cruz Biotechnology, #sc7382). These were mixed in 1× PBS, 5% *w*/*v* nonfat dried milk, and 0.1% Tween-20 at 4 °C overnight. To ensure that blots were loaded with equal amounts of proteins, they were also probed with antibodies against β-actin protein for cytosolic fraction (1:500; Santa Cruz Biotechnology Heidelberg, Germany) or lamin A/C for nuclear fraction (1:500 Sigma-Aldrich, Milan, Italy). Signals were examined with an enhanced chemiluminescence (ECL) detection system reagent according to the manufacturer’s instructions (Thermo, Monza, Italy). The relative expression of the protein bands was quantified by densitometry with Bio-Rad ChemiDoc^TM^ XRS^+^ software (Version 6.0.1, Milan, Italy) and standardized to the β-actin and lamin A/C levels [71,72,73,74,75]. 

### 2.5. Testosterone Assay

For testosterone assessment, blood samples were collected from the heart. The serum was separated from blood with 15 min of centrifugation at 3000× *g* and stored at −20 °C for analysis. Serum testosterone levels were measured in accordance with the manufacturer’s instructions (Mouse Testosterone ELISA Kit, Bioassay, Cat. #MBS702281, San Diego, CA, USA). The amount of testosterone is expressed as nmol/L. All samples were analyzed in duplicate.

### 2.6. Histopathological Evaluation 

Bladder and testes were dehydrated, embedded in paraffin, and stained in hematoxylin/eosin (H/E), as previously described [69]. Testicular damage was assessed considering Johnsen’s score (JS), ranging from 0 (no seminiferous epithelial cells; tubular sclerosis) to 10 (full spermatogenesis) [63,76]. Bladder damage was assessed on a scale from 0 (no inflammation) to 5 (severe inflammation) as previously described. The degree of bladder fibrosis was evaluated by Masson’s trichrome method according to the manufacturer’s protocol (Bio-Optica, Milan, Italy). For staining, sections from each mouse were observed using a Leica DM6 microscope (Leica Microsystems SpA) and scored in a blinded fashion [77,78].

### 2.7. Evaluation of Tissue Lipid Peroxidation

Lipid peroxidation levels were assessed via two methods: thiobarbituric acid reactive substance (TBARS) formation in the testes and malondialdehyde (MDA) levels in the bladder [77,79].

### 2.8. Assessment of Tissue Antioxidant Activity

SOD and CAT activity and GSH concentration were examined as previously described in other works [19,80,81,82].

### 2.9. Terminal Deoxynucleotidyl Nick-End Labeling (TUNEL) Assay

TUNEL staining for apoptotic cell nuclei and DAPI staining for all cell nuclei were performed in lung sections as described previously [31,71,72,73,74,75]. The index of apoptosis is expressed as the number of positively stained apoptotic cells over the total number of cells counted, multiplied by 100% [83,84,85,86,87,88,89]. 

### 2.10. Cytokine Levels

Homogenates of testes and the bladder were prepared according to the manufacturer’s instructions. Supernatants were used for the estimation of TNF-α, IL-1β, and IL-6 using ELISA kits [32,90,91,92,93,94,95].

### 2.11. Materials

Unless otherwise stated, all compounds were purchased from Sigma-Aldrich (Milan, Italy). 

### 2.12. Statistical Evaluation

All values are expressed as mean ± standard error of the mean (SEM) of N observations. For in vivo studies, N represents the number of animals used. The results were analyzed by one-way ANOVA followed by a Bonferroni post-hoc test for multiple comparisons. A *p*-value less than 0.05 was considered significant.

## 3. Results

### 3.1. Açai Berry Limitis CYP-Induced Macroscopic and Microscopic Alterations 

**Figure 1:**
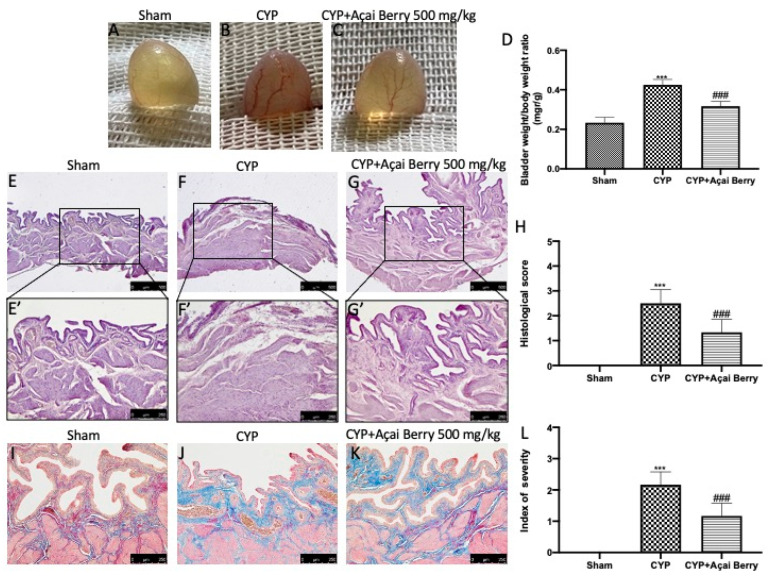
Impact of açai berry on CYP-induced bladder alteration. Macroscopic bladder in (**A**) sham, (**B**) CYP, and (**C**) açai berry treatments. (**D**) Bladder weight. Histological evaluation of bladder stained with H/E in (**E**,**E′**) sham, (**F**,**F′**) CYP, and (**G**,**G′**) açai berry treatments. (**H**) Histological score. Masson’s trichrome of (**I**) sham, (**J**) CYP, and (**K**) açai berry treatments. (**L**) Index of severity. *** *p* < 0.001 vs. sham; ### *p* < 0.001 vs. CYP.

After CYP injection, we found macroscopic edema and hyperemia as well as increased weight compared to sham animals (Figure 1A,B,D,L). Histopathological examination of the bladder showed important alterations after CYP injection. In particular, with H/E we observed epithelial denudation, cellular infiltration, and edema with moderate inflammatory exudates in the mucosa (Figure 1E,E′,F,F′,H). Additionally, by Masson’s trichrome we found a significant increase in collagen deposition after CYP injection compared to the control group (Figure 1I,J). After daily oral administration of açai berry, we found a significant decrease in macroscopic damage (Figure 1C) and weight (Figure 1D) compared to the CYP group as well as a decrease in histopathological alteration (Figure 1G,G′,H) and collagen deposition (Figure 1K).

### 3.2. Effect of Açai Berry Administration on CYP-Induced Bladder Oxidative Stress and Lipid Peroxidation 

**Figure 2:**
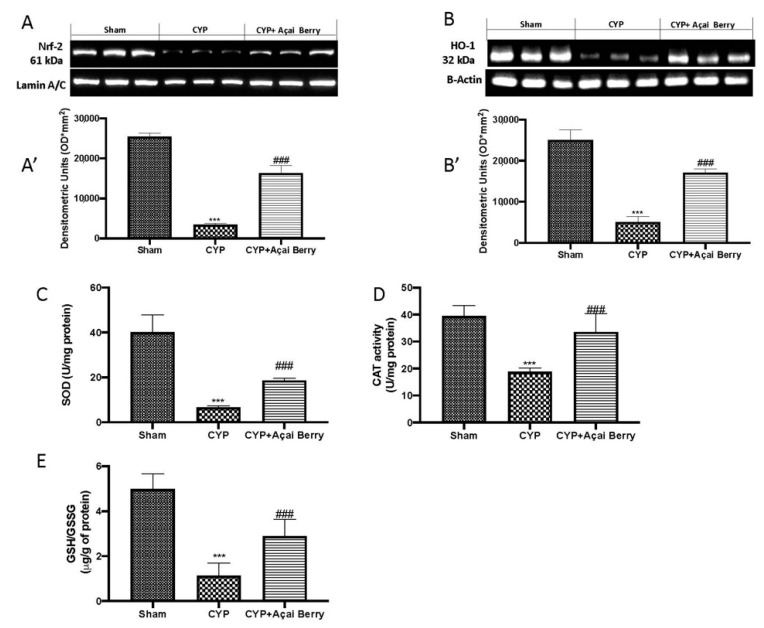
Effect of açai berry administration on CYP-induced bladder oxidative stress. (**A**) Western blot analysis and (**A′**) densitometric analysis of Nrf-2; (**B**) Western blot analysis and (**B′**) densitometric analysis of HO-1; (**C**) SOD; (**D**) CAT; (**E**) GSH/GSSG. *** *p* < 0.001 vs. sham; ### *p* < 0.001 vs. CYP.

To determine whether açai berry could modulate CYP-induced oxidative stress, we investigated Nrf-2 pathways in the bladder. By Western blots, we observed a significant perturbation in Nrf-2 expression (Figure 2A,A′) and HO-1 (Figure 2B,B′). These increases in oxidative stress also induced a critical perturbation in SOD (Figure 2C), CAT (Figure 2D), and GSH/GSSG (Figure 2E) activity, as determined by ELISA. However, after açai berry administration at a dose of 500 mg/kg, we found a significant improvement in endogenous antioxidant defense. 

### 3.3. Effect of Açai Berry Administration on Apoptosis Pathways after CYP

**Figure 3:**
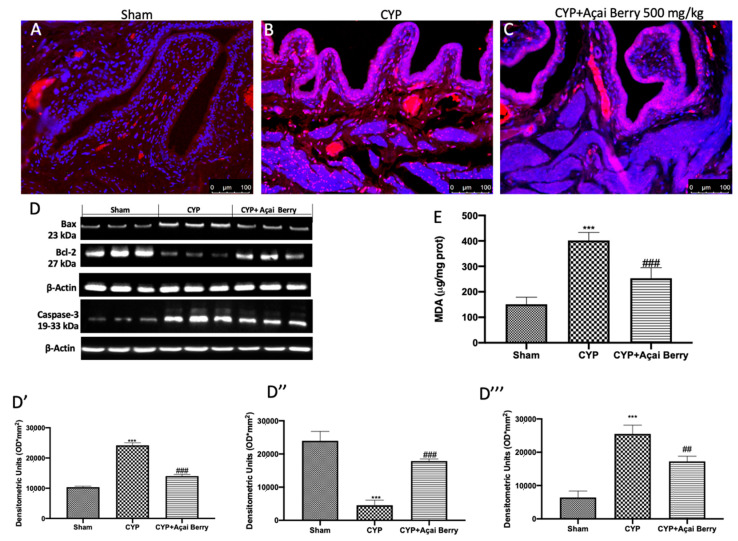
Açai berry modulates CYP-induced apoptosis pathways. TUNEL staining of (**A**) sham, (**B**) CYP, and (**C**) açai berry treatments. Western blot analysis of Bax, Bcl-2, and caspase 3 (**D**) and densitometric analysis (respectively, **D′**,**D″**,**D‴**). (**E**) MDA levels. *** *p* < 0.001 vs. sham; ## *p* < 0.01 vs. CYP; ### *p* < 0.001 vs. CYP.

Injection of CYP induced serious damage to the bladder, leading to apoptosis. By TUNEL, staining we found that after CYP injection (Figure 3B) there was an increase in apoptotic cells compared to the sham group (Figure 3A); however, after açai berry administration, we noticed a decrease by co-staining with DAPI-TUNEL (Figure 3C). To further analyze cell death, we performed Western blot analysis for Bax, Bcl-2, and cleaved caspase 3. After CYP injection, we found a significant increase in BAX expression (Figure 3D,D′) and cleaved caspase 3 expression (Figure 3D,D‴), and, on the contrary, a decrease in Bcl-2 (Figure 3D,D″). Additionally, we investigated lipid peroxidation in bladder after CYP injection by MDA levels. As shown in Figure 3E, we found a significant decrease in CYP-induced MDA levels after açai berry administration.

### 3.4. Effect of Acai Berry Administration on Cytokyne Storm in Bladder and Testes after CYP

**Figure 4:**
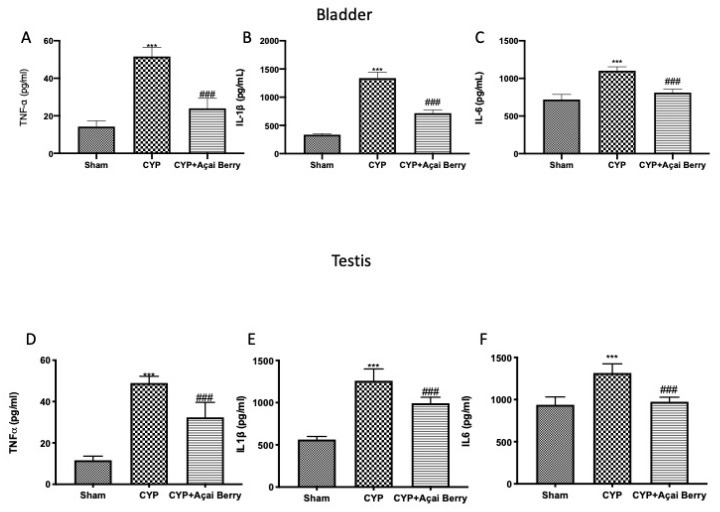
Effect of açai berry on cytokine storm. ELISA quantification of (**A**) TNF-α, (**B**) IL-1β, and (**C**) IL-6 levels in bladder and (**D**) TNF-α, (**E**) IL-1β, and (**F**) IL-6 levels in testes. *** *p* < 0.001 vs. sham; ### *p* < 0.001 vs. CYP.

Cytokine storm may be the connection point between bladder and testis inflammation after CYP. For this reason, we investigated by ELISA the levels of TNF-α, IL-1β, and IL-6 in bladder (Figure 4A–C) and testes (Figure 4D–F). In agreement with the literature, we found that CYP injection induced a significant increase in cytokine levels in bladder and testes. However, after açai berry administration, the levels in both organs were significantly diminished. 

### 3.5. Effect of Açai Berry Administration on Sperm Parameters and Testosterone Levels after CYP 

**Figure 5:**
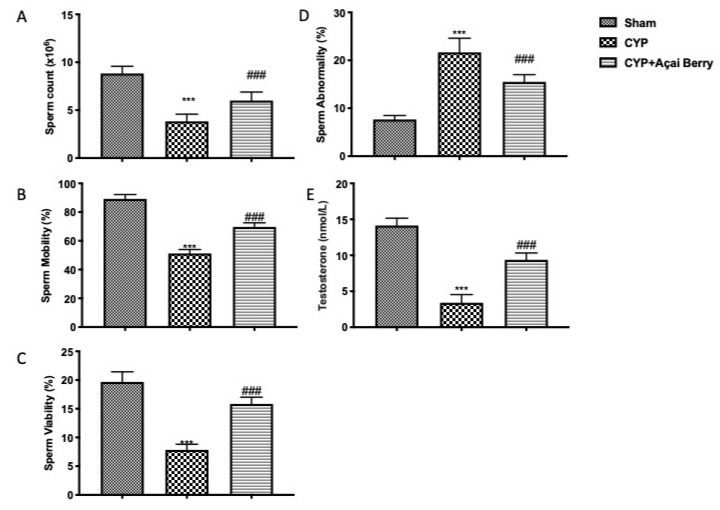
Effect of açai berry administration on sperm parameters and testosterone levels after CYP administration: (**A**) sperm count, (**B**) sperm motility, (**C**) sperm viability, (**D**) sperm abnormality, (**E**) serum testosterone level. *** *p* < 0.001 vs. sham; ### *p* < 0.001 vs. CYP.

Administration of CYP resulted in significant modification of sperm parameters. We specifically saw a decline in sperm viability (Figure 5A), motility (Figure 5B), and count (Figure 5C); we also noticed an increase in sperm abnormalities (Figure 5D). The considerable difference in testosterone levels between CYP-treated and control groups further demonstrated the damage to the testicles (Figure 5E). All sperm parameters considered, as well as testosterone levels, were restored after açai berry administration to almost the same level of the control group. 

### 3.6. Effect of Açai Berry on Histological Alteration in Testes after CYP

**Figure 6:**
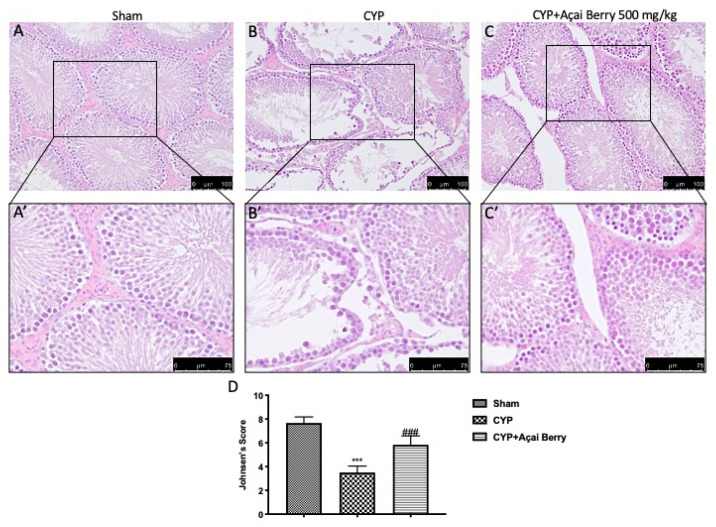
Effect of açai berry administration on testis architecture. Histological photographs of testicular tissue and relative higher magnification in (**A,A′**) sham, (**B,B′**) CYP, and (**C,C′**) açai berry treatments. (**D**) Graphical representation of Johnsen’s score. *** *p* < 0.001 vs. sham; ### *p* < 0.001 vs. CYP.

The information from the sperm analysis inspired us to carry out more research on the morphological changes resulting from CYP treatment. The control group (Figure 6A,D) exhibited the typical architecture of the testes, with distinct seminiferous tubules at various stages of spermatogonial cells. Additionally, spermatozoa and interstitial cells could be seen inside a lumen. Leydig cells and Sertoli cells, both of which have distinct nuclei and substantial cytoplasm, were also discernible in various sections. Following CYP, testicular tissues showed a reduction of spermatozoa in the lumen and disordered spermatogenic cells with fewer spermatids. Additionally, it was found that the epithelial wall of the seminiferous tubules was disturbed and injured, and there was more interstitial edema (Figure 6B,D). Testicular tissue looked repaired after the administration of 500 mg/kg açai berry, with a normal amount of spermatozoa, decreased edema, and reduced luminal disruption (Figure 6C,D).

### 3.7. Açai Berry Administration Improved Endogenous Oxidative Defense in Testes 

**Figure 7:**
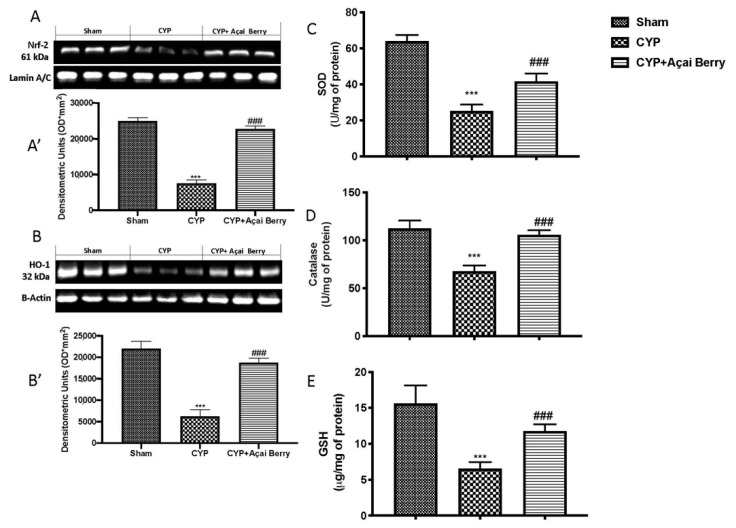
Açai berry administration improves endogenous oxidative defense. (**A**) Western blot of testicular tissue for Nrf2 and (**A’**) densitometric analysis; (**B**) Western blot of HO-1 and (**B’**) densitometric analysis. Analysis of (**C**) SOD, (**D**) catalase, and (**E**) GSH in testis. *** *p* < 0.001 vs. sham; ### *p* < 0.001 vs. CYP.

The findings from Western blot examination demonstrated a significantly lower level of Nrf2 expression in CYP-injected mice than in sham animals (Figure 7A,A′). We also investigated the expression of HO-1 using Western blotting, because it is an Nrf2-regulated gene that is essential for preserving oxidant/antioxidant balance. Similar to Nrf2, HO-1 was diminished following CYP (Figure 7B,B′). Following oral administration of açai berry at a dose of 500 mg/kg, these decreases were entirely reversed. Additionally, we used an ELISA kit to evaluate endogenous antioxidant system markers and discovered that açai berry at a dose of 500 mg/kg was sufficient to considerably counteract CYP-induced decreases, nearly restoring physiological levels of SOD (Figure 7C), catalase (Figure 7D), and GSH (Figure 7E).

### 3.8. Açai Berry Administration Limits CYP-Induced Apoptosis and TBARS in Testes 

**Figure 8:**
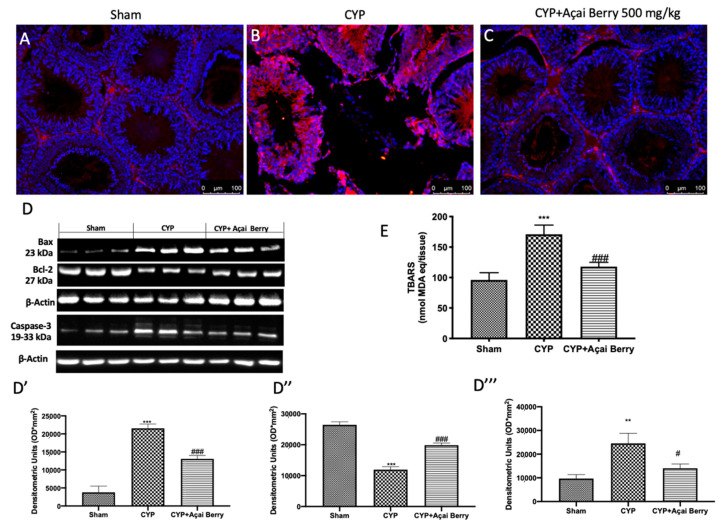
Açai berry administration limits CYP-induced apoptosis and TBARS in testes. Representative TUNEL photos of (**A**) sham, (**B**) CYP, and (**C**) açai berry treatments. Western blots and quantification of testicular tissue for (**D**) Bax, Bcl-2, and caspase 3 and respectively densitometric analysis (**D′**,**D″**,**D‴**). (**E**) TBARS quantification in testes. ** *p* < 0.01 vs. sham; *** *p* < 0.001 vs. sham; # *p* < 0.05 vs. CYP; ### *p* < 0.001 vs. CYP.

In line with previous results found in the bladder, we discovered a large rise in TBARS in the CYP-induced group compared to the control group (Figure 8E); however, there was a significant decrease after oral administration of açai berry at a dose of 500 mg/kg. It is commonly known that apoptosis and oxidative stress are strongly related. By TUNEL staining, we found that there was a significant increase in apoptotic cells in testes of the CYP-injected group (Figure 8B) compared to the sham group (Figure 8A). On the other hand, after açai berry administration we noticed a significant decrease in cell death (Figure 8C). By using Western blot, we additionally looked at the expression of Bax, cleaved caspase 3, and Bcl-2 and discovered that, in comparison to control groups, Bax (Figure 8D,D′) and cleaved caspase 3 (Figure 8D,D‴) expression was higher in CYP-induced testicular injury. However, açai berry was able to greatly reduce Bax and cleaved caspase 3 expression and increase Bcl-2 expression (Figure 8D,D″).

## 4. Discussion

CYP, a bifunctional cytotoxic alkylating agent from the nitrogen mustard drug class, is used to treat a range of cancers as well as organ transplant rejection and autoimmune diseases [96,97,98,99]. It is extremely poisonous to both people and animals, especially the liver, urinary tract, and reproductive organs, despite having a wide range of medical benefits [3,35,100,101,102,103,104,105,106,107]. CYP is quickly processed in aldophosphamide mustard and acrolein, which inhibit cellular DNA synthesis [108]. Acrolein induces toxicity and substantial oxidative stress that result in the loss of cell lipids, proteins, and DNA [109]. The unexpected toxicity of CYP in cells limits its therapeutic efficacy. Therefore, it is essential to avoid CYP-induced cell DNA breakage in therapeutic settings [110]. 

The idea that “food is the best medicine” refers to the importance of dietary components, especially those that support good health [111]. 

The fruit from the tropical palm tree of the genus *Euterpe*, which is native to South America and is known as açai, is a new food of interest to scientists. Researchers have been investigating the fruit of *Euterpe oleracea* because it has a high antioxidant content compared to other fruits and berries. It has been determined from research on the chemical makeup of açai pulp that it includes several phytochemicals with physiological activity. Numerous studies have shown that açai berries have beneficial effects, such as restoring calcium homeostasis and mitochondrial function, preventing the formation of toxic protein aggregates, and providing antioxidant and anti-inflammatory activity [112,113,114,115,116,117,118,119].

Here, we demonstrate for the first time in a combined model of CYP-induced urogenital impairment in mice that treatment with açai berry may have a positive effect. It is generally known that CYP-induced cystitis causes oxidation and inflammation in the bladder. In our study, after one injection of CYP at a dose of 200 mg/kg, we noticed a significant increase in macroscopic and microscopic damage, with a perturbation in the oxidant–antioxidant balance. After açai berry administration at a dose of 500 mg/kg, we found that the histological alterations were significantly reduced, and the NRF-2 pathway was significantly restored, with decreased oxidative stress, due to the enhancement of physiological antioxidant enzymes.

As described above, acrolein exerts a toxic effect on cells by several mechanisms, leading to apoptosis and cell death. In our work, we demonstrated by TUNEL staining that açai berry is able to counteract CYP-induced apoptosis in the bladder [120]. 

Connected to oxidative stress and apoptosis is the activation of the inflammatory response, with the release of pro-inflammatory cytokines in the bladder and the testes. In our work, we found that the CYP-induced release of TNF-α, IL-1β, and IL-6 was significantly reduced after açai berry administration. The transient interference with the healthy functioning of the male reproductive system and testosterone levels that CYP creates is well recognized [121].

Several histological changes were presumably connected to a decline in sperm viability. CYP leads to perivascular fibrosis, spermatogonia degeneration and vacuolation, diminished spermatocytes and germ cells, irregular seminiferous tubules, diminished seminiferous epithelial layers, severe maturation arrest, and decreased size and number of seminiferous tubules [97,122,123]. In our investigation, açai berry was able to raise testosterone levels following CYP treatment as well as restore sperm counts and vitality. The histological structure of CYP-treated mice revealed decreased seminiferous tubule width, decreased numbers of germinal cells, tubule atrophy, Sertoli cell vacuolization, interstitial edema, and congestion [63]. Açai berry at a dose of 500 mg/kg was able to limit CYP-induced histological alterations.

Low levels of ROS, which are necessary for numerous physiological processes including capacitation, hyperactivation, and sperm-oocyte fusion, have been shown to be produced by spermatocytes and spermatids. Because of the high levels of polyunsaturated fatty acids in their plasma membranes and the low levels of scavenging enzymes in their cytoplasm, spermatozoa are particularly susceptible to harm from excessive ROS. Male germ cell proliferation and development from diploid spermatogonia to mature haploid spermatozoa via meiosis is known as spermatogenesis, which is a complex process [124,125]. 

The testes have developed a sophisticated antioxidant system that consists of enzymes and free radical scavengers to reduce this risk. Excessive ROS is mostly eliminated by endogenous antioxidant enzymes including SOD, GSH, and CAT or by Nrf2 activation [126,127]. 

As described above for the bladder, when CYP was used, it was found that the levels of Nrf2 in the testes were decreased. Additionally, CYP treatment interfered with the normal antioxidant response and molecules in the downstream Nrf2 pathway, including HO-1 and SOD [128]. It is noteworthy that açai berry greatly increased the amounts of Nrf2 and HO-1 and significantly enhanced the physiological antioxidant response by increasing catalase and GSH activity. 

Peroxides, alcohol, and lipidic aldehydes can be produced as by-products when ROS attack the unsaturated bonds in membrane lipids during the autocatalytic process. As a result of the oxidative degradation of polyunsaturated fatty acids, an increase in free radicals in cells can cause lipid peroxidation in cell membranes, leading to apoptosis [63,129,130]. Oral administration of açai berry at a dose of 500 mg/kg was sufficient to counteract lipid peroxidation, as demonstrated by the decrease in TBARS and apoptosis by TUNEL staining and molecular investigation of Bax and Bcl-2 expression.

## 5. Conclusions

In our study we demonstrate for the first time that açai berry, by modulating oxidative stress and inflammation, inhibiting the release of pro-inflammatory mediators, and diminishing apoptosis, could be useful as a dietary supplement to counteract CYP-induced urogenital toxicity in patients. A limitation of this study is the possible interaction between CYP and açai berry. Clearly, more studies are needed to investigate whether açai berry has anticancer or immunomodulatory effects against CYP. For this reason, a follow--up study is needed to determine the effects of açai berry and CYP, used singly and in combination, on tumor growth in live animals. If such treatment turns out to have a beneficial effect, combined treatment would be warranted in a clinical setting.

## Data Availability

The data used to support the findings of this study are available from the corresponding author upon request.

## Supplementary Materials

The following supporting information can be downloaded at: https://www.mdpi.com/article/10.3390/antiox11122355/s1, Figure S1: experimental design.
