# Supplementary figures and images for: Açai Berry Attenuates Cyclophosphamide-Induced Damage in Genitourinary Axis-Modulating Nrf-2/HO-1 Pathways
Source: Antioxidants (Basel). 2022 Nov 28;11(12):2355. doi: 10.3390/antiox11122355 (PMC9774754; doi:10.3390/antiox11122355)

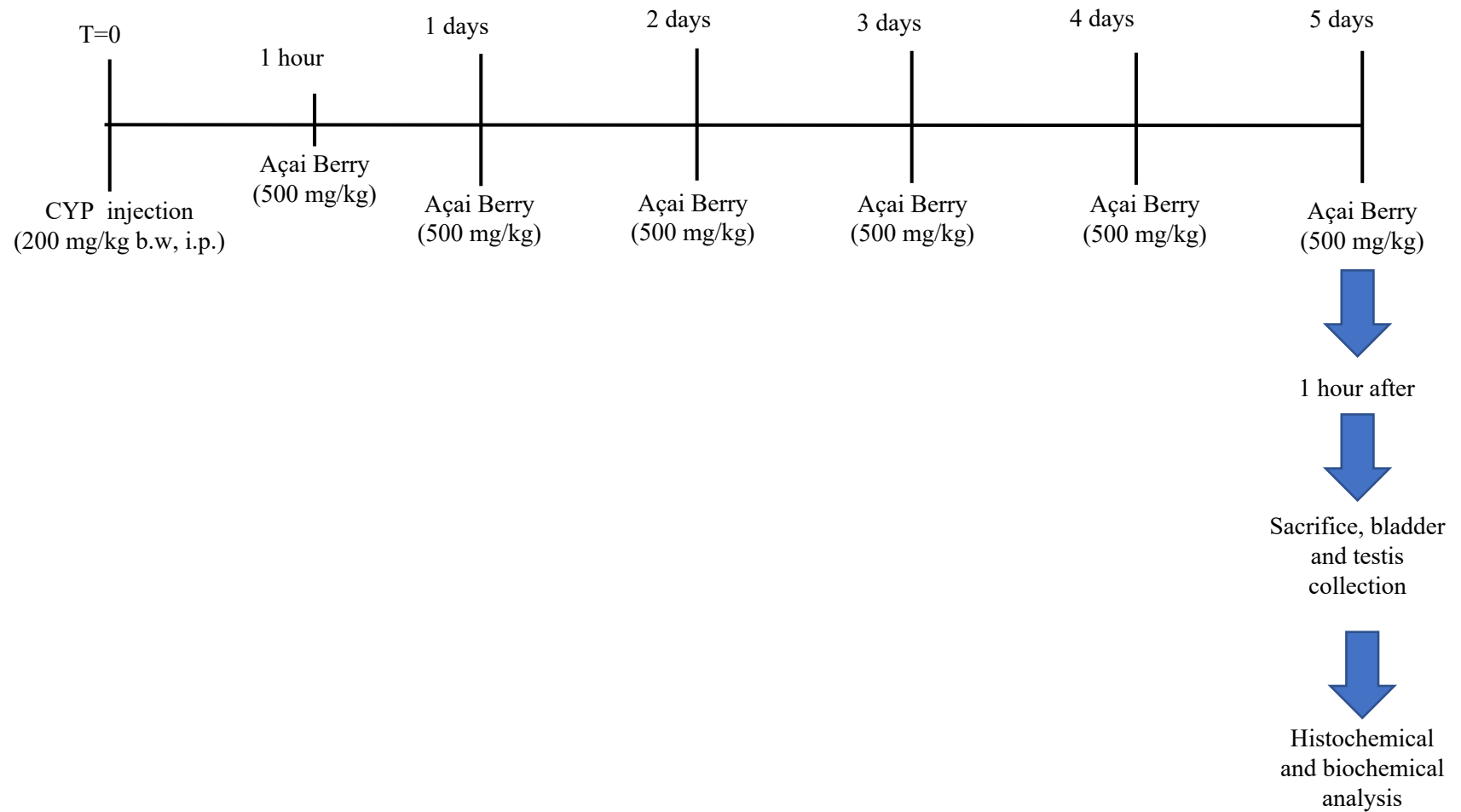

Figure S1: Experimental design.

Supplement: Supplementary file 1 [file antioxidants-11-02355-s001.zip › antioxidants-2019011-supplementary.pdf]
